# Concomitant sacroiliac joint abnormalities in patients with femoroacetabular impingement

**DOI:** 10.1007/s00264-025-06484-6

**Published:** 2025-03-29

**Authors:** Sonia E. Ubong, Teresa Clode Araújo, Zaki Arshad, Vikas Khanduja

**Affiliations:** 1 University of Cambridge, Cambridge, UK; 2 Hospital de Cascais Dr. José de Almeida, Alcabideche, Portugal; 3 University Hospitals of Leicester NHS Trust, Leicester, UK; 4 Addenbrooke’s Hospital, Cambridge, UK

**Keywords:** Sacroiliac joint, Femoroacetabular impingement, Axial spondyloarthritis, Scoping review

## Abstract

**Purpose:**

Despite the increasing understanding of femoroacetabular impingement (FAI), the impact of specific patient characteristics, including inflammatory pathologies like axial spondyloarthritis (axSpA), on its pathophysiology and clinical outcomes following treatment remains inadequately defined. Therefore, the purpose of this scoping review was to evaluate the relationship between FAI and sacroiliac (SI) joint abnormalities and FAI and axSpA.

**Methods:**

The study was conducted following the framework established by Arksey and O’Malley and Levac et al., adhering to the PRISMA scoping review extension checklist. A systematic search was performed across MEDLINE, EMBASE, and Cochrane Library databases for articles published until August 2024. A total of 120 articles were screened and eight finally met the inclusion criteria.

**Results:**

The review analysed data from the eight retrospective studies with a total of 1,723 patients. We found that the prevalence of SI joint abnormalities in patients with FAI can be as high as 25–28%. Furthermore, the prevalence of FAI morphology in patients with axial spondyloarthritis can be as high as 20–37%. Finally, patients undergoing hip arthroscopy for FAI with axSpA and/or SI joint abnormalities have lower postoperative outcome scores reported in comparison with those patients who do not have these comorbidities.

**Conclusion:**

Over a quarter of patients with FAI can have concomitant radiographic SI joint abnormalities. We cannot overemphasise the importance of assessing the spine, specifically the SI joint, and ruling out symptoms emanating from the SI joint in all patients with FAI. There is clearly a knowledge gap in understanding the underlying pathophysiology linking FAI and axSpA. We require further research to elucidate the underlying mechanisms of this relationship, standardise evaluation methods, and explore long-term outcomes in this cohort of patients.

## Introduction

Despite the exponential rise in hip arthroscopy in patients with femoroacetabular impingement syndrome (FAI), the role of specific patient characteristics on the pathophysiology of FAI remains unclear [1, 2]. FAI is characterised by the presence of hip pain, restricted range of motion of the hip and abnormal morphology on imaging of the hip joint [3, 4]. Consequently, it has now been recognised as a significant risk factor for the onset of progressive degenerative changes and the development of early osteoarthritis (OA) in the non-dysplastic hip [3, 5, 6]. While hip arthroscopy is the established treatment of choice, not all patients with FAI benefit equally from this procedure [7]. For some patients, hip arthroscopy may not only fail to alleviate symptoms but could potentially lead to complications [8, 9] or unnecessary surgical intervention. This lack of universal success highlights a critical gap in our understanding of FAI and its management. Importantly, the focus on FAI morphology may sometimes overshadow other underlying conditions that could be the true source of a patient’s hip pain. Therefore, it is crucial to investigate factors contributing to the variability in treatment outcomes [10]. Recent findings suggest that the aetiology of hip pain in FAI may be more complex than previously thought, potentially involving systemic factors beyond local joint mechanics, with emerging research exploring connections to conditions such as axial spondyloarthritis (axSpA) [11–13].

AxSpA is a chronic and progressive inflammatory condition which generally affects young individuals, typically starting in late adolescence. A diagnosis of axSpA is commonly considered in the presence of chronic back pain [14] and is associated with progressively worsening stiffness, pain, and impaired physical function [15]. A hallmark feature of axSpA is inflammation of the sacroiliac (SI) joints, which can lead to significant pain and structural changes in these joints over time. It is important to note that in axSpA, large joints including the hip can be affected by the inflammatory process itself, with or without FAI morphology. The prevalence of hip involvement in axSpA has been reported to range from 24 to 36% across various studies, emphasising the importance of distinguishing between inflammatory hip disease and mechanical impingement in these patients. As with axSpA FAI most commonly affects young adults, though through different mechanisms. Patients with FAI typically present with painful clicking, locking or instability but may also present with symptoms that overlap with other musculoskeletal conditions of the hip, pelvis, and lumbar spine [16]. Anterior groin pain is a predominant feature of FAI and often affiliated with intra-articular pathology and labral tears, however, posterior hip pain in these patients is associated with SI joint abnormalities [17, 18]. There are several patients presenting with clinical features of FAI and concomitant low back pain [19, 20] which may well be secondary to axSpA. It is therefore crucial to investigate factors responsible for outcomes not being universally good and to consider a broader differential diagnosis before proceeding with surgical intervention.

Nonetheless, a further in-depth scrutiny of the relationship between axSpA and FAI is required. Therefore, the purpose of this scoping review was to evaluate the relationship between FAI and SI joint abnormalities and FAI and axSpA.

## Methods

The scoping review was performed as per the methodological framework proposed by Arksey and O’Malley and Levac et al. [21, 22] and was guided by the Preferred Reporting Items for Systematic Reviews and Meta-Analyses (PRISMA) scoping review extension checklist [23]. A scoping study methodology was chosen to specifically map the existing research on this topic and identify knowledge gaps in the literature to accurately guide future research.

### Search strategy

A refined search strategy was conducted in the following electronic databases for articles published from launch of database (stated in brackets) to August 2024: MEDLINE (1966), EMBASE (1980), and The Cochrane Library (1996). Briefly, the search strategy included synonyms, related terms, and subtypes of FAI and axSpA as follows: (femoroacetabular impingement OR FAI OR cam OR pincer) AND (spondyloarthritis OR spondylitis OR axial spondyloarthritis OR axSpA OR ankylosing spondylitis (AS) OR sacroiliitis OR sacroiliac joint disease OR hip-spine syndrome.) There were no restrictions on language or the publication year.

### Study selection

The study selection process was conducted in three stages: title screening, abstract review, and full-text assessment. Initially, the principal investigator performed an extensive title screening based on the review’s eligibility criteria. Subsequently, two independent researchers screened titles and abstracts to remove irrelevant and duplicate articles. The remaining full-text articles were then assessed according to pre-determined eligibility criteria and shared among the review team.

To be eligible for inclusion, studies had to report on any aspect of the relationship between FAI and axSpA, including related pathologies such as SI joint pathology. All included studies were required to have FAI diagnosis confirmed by radiological investigation (X-ray, CT or MRI), while axSpA-related conditions were to be diagnosed according to established clinical and imaging criteria. Studies were excluded if they were case reports with fewer than ten patients or were non-original research articles such as commentaries or editorials.

Two researchers independently conducted the full-text screening of the selected papers. Any discrepancies in decisions regarding study inclusion were resolved through discussion or, when necessary, consultation with a third senior reviewer. Articles deemed ineligible were removed and the reasons for exclusion were documented. To ensure comprehensive coverage, a manual search of the reference lists of all included studies was conducted to identify any additional relevant articles that may have been missed in the initial database search.

### Data extraction from included studies

The data of interest from each article was thoroughly reviewed by all authors. Data extraction was performed using the full text of the articles and included the following information: author (s), year, study design, patient demographics, sample sizes, details regarding associated axial pathology and FAI and primary outcomes. One author extracted the data, and another validated them to ensure accuracy. Descriptive analysis of the data is presented.

## Results

**Fig. 1:**
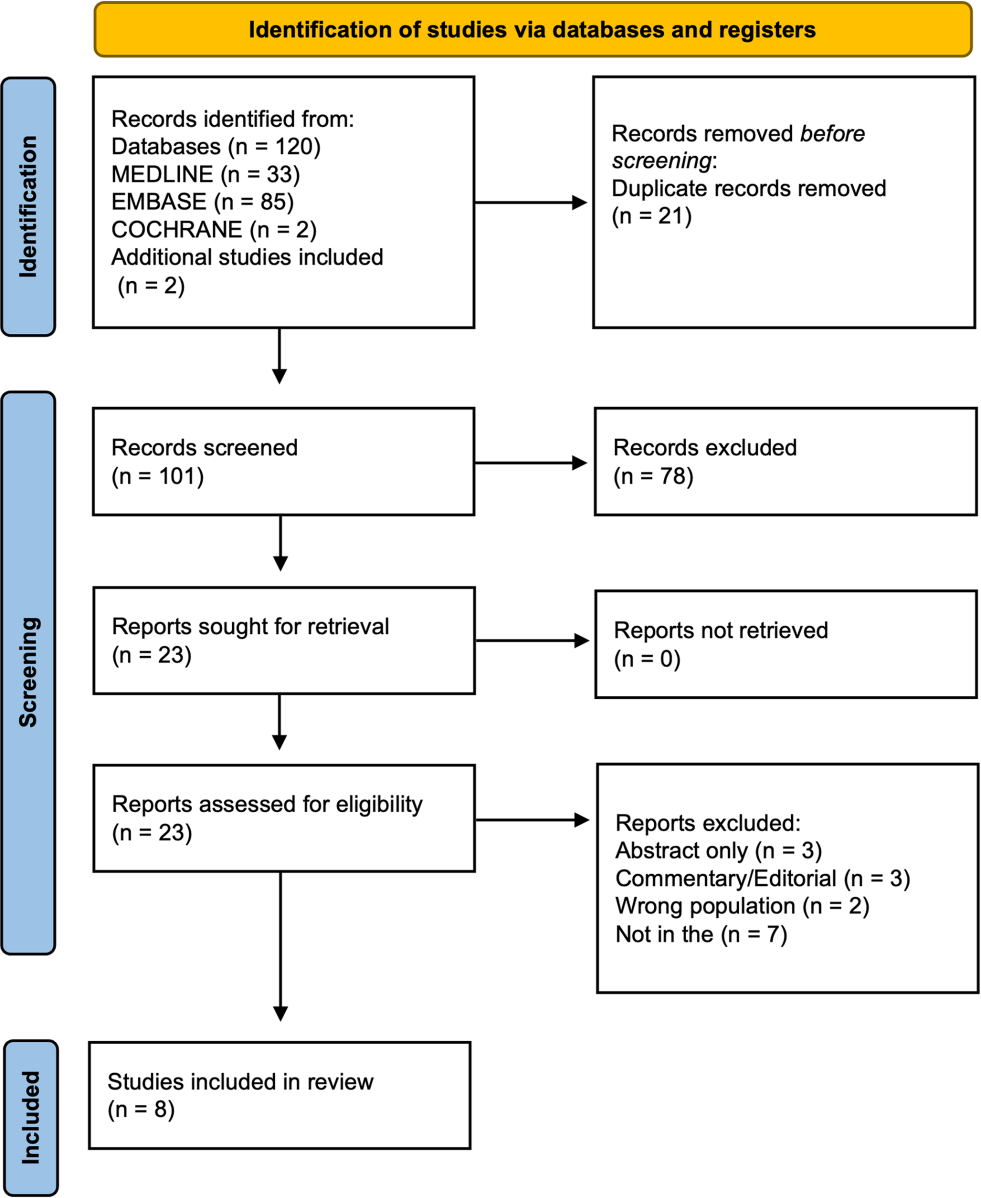
PRISMA (Preferred Reporting Items for Systematic Reviews and Meta-Analyses) flowchart of article selection and screening. n, number articles excluded based on title and abstract review by 2 authors

The initial query of the Medline, Embase, and Cochrane databases resulted in 120 articles. After duplicates were removed and two additional publications were added through manual searching, there were 101 studies. The titles, abstracts, and keywords of the remaining articles were reviewed to determine initial relevancy, and 23 articles remained. Full-text reviews of the remaining articles were conducted, and 8 of these articles met the inclusion criteria. Figure 1 provides a detailed illustration of the study identification and selection process, adhering to the PRISMA guidelines.

### Demographic characteristics

Demographic and methodological characteristics of the included studies are summarised in Table 1. Our review encompassed eight retrospective studies published between 2013 and 2024, with sample sizes ranging from 28 to 743 participants with a total of 1,723 participants across all studies. Study periods spanned from 2004 to 2022, with durations varying from two to 17 years. The mean age of participants ranged from 16.1 years [12] in a paediatric cohort to 44.6 years [24] with most studies focusing on adult populations. Gender distribution varied considerably across studies, with male participation ranging from 18.7 to 76.1%. Associated spinal/pelvic pathologies included SI joint abnormalities, AS, sacroiliitis, axSpA, and SI joint pain. SI joint abnormalities were the most frequently reported associated pathology, featured in four studies [18, 24–26]. Primary outcome measures varied across studies but generally included FAI prevalence, association with sacroiliitis or SI joint abnormalities, and patient-reported outcomes and pain scores.

**Table 1:** Demographic characteristics

Authors (Year)	Study Type	Study Design	Study Period	Number of Participants	Mean age, years	Male to Female Ratio (%)	Primary Outcomes Measures
Ferguson et al. (2021)	Case-control	R	2004–2013	28	44.6	11 (39.3)	Prevalence of SI joint abnormalities
Horner et al. (2022)	Case-control	R	2012—2018	73	35.6	14 (18.7)	FAI prevalence, association with sacroiliitis or sacroiliac joint abnormalities, and patient-reported outcomes and pain scores
Ido et al. (2023)	Cross-sectional	R	2005–2022	67	36.5	51 (76.1)	FAI prevalence, association with sacroiliitis
Krishnamoorthy et al. (2019)	Case-control	R	2012–2016	743	33.3	236 (31.8)	Prevalence of SI joint abnormalities, patient-reported outcomes, pain scores
Lee et al. (2015)	Case series	R	2004–2012	384	35.9	288 (75.0)	FAI prevalence, association with sacroiliitis
Morgan et al. (2013)	Case series	R	2006–2009	30	NR	9 (30.0)	FAI prevalence
Seth et al. (2020)	Case-control	R	2016–2018	90	16.1	34 (37.8)	Prevalence of inflammatory conditions
Tosun et al. (2021)	Case-control	R	NR	158	41.5	87 (55.1)	FAI prevalence

*AS*: ankylosing spondylitis; *axSpA*: axial spondyloarthritis; *FAI*: femoroacetabular impingement *NR*: not reported; *R*: retrospective; *SI*: sacroiliac

### Relationship between FAI and the sacroiliac joint

Two studies specifically examined the prevalence of SI joint abnormalities in patients with FAI (Tables 2 and 3). Krishnamoorthy et al., in a comprehensive cohort study utilising multiple imaging modalities (X-ray, CT, MRI), reported a prevalence of SI joint abnormalities in 25.2% of patients with FAI [18]. Ferguson et al. corroborated these findings, observing a slightly higher prevalence of 28.6% in a more specific cohort of FAI patients with labral ossification, using X-ray and CT imaging [24]. Notably, Krishnamoorthy et al. further elucidated that patient with concomitant SI joint abnormalities demonstrated significantly lower functional outcome scores and higher pain scores at follow-up compared to those without SI joint involvement. Morgan et al. approached the association from a different perspective, reporting that 77% of patients presenting with SI joint pain exhibited at least one radiographic abnormality consistent with FAI morphology [26].

**Table 2:** Summary of study characteristics

Authors (Year)	Type of morphology investigated in study	Radiographic Definition of FAIS	Associated Spinal/Pelvic Pathology	Primary Outcomes Measures
Ferguson et al. (2021)	Labral ossification in FAI	Cam: Alpha angle > 55 Pincer: Elevated lateral center-edge angle (> 40)	SI joint abnormalities	Prevalence of SI joint abnormalities
Horner et al. (2022)	Cam	Cam: Alpha angle > 57°	SI joint pain	FAI prevalence, association with sacroiliitis or sacroiliac joint abnormalities, and patient-reported outcomes and pain scores
Ido et al. (2023)	Cam and pincer	Cam: presence of pistol grip configuration Pincer: CE angle > 40 or CE angle > 25 and the presence of COS	AS, Sacroiliitis	FAI prevalence, association with sacroiliitis
Krishnamoorthy et al. (2019)	Not specified	NS	SI joint abnormalities	Prevalence of SI joint abnormalities, patient-reported outcomes, pain scores
Lee et al. (2015)	Cam, pincer, combined	Cam: Alpha angle > 55 Pincer: Elevated lateral CE angle (> 39)	AS	FAI prevalence, association with sacroiliitis
Morgan et al. (2013)	Cam and pincer	Cam: asphericity > 2 mm from the anterior femoral neckline Pincer: Elevated lateral CE angle (> 40)	SI joint disease	FAI prevalence
Seth et al. (2020)	Cam	Not specified	AS	Prevalence of inflammatory conditions
Tosun et al. (2021)	Cam	Cam: presence of pistol grip configuration	axSpA	FAI prevalence

*AS*: ankylosing spondylitis; *axSpA*: axial spondyloarthritis; *CE*: centre edge; *COS*: cross over sign; *FAI*: femoroacetabular impingement *NS*: not specified; *R*: retrospective; *SI*: sacroiliac

**Table 3:** Sacroiliac joint abnormalities in FAI patients

Authors (Year)	Total Patients	Patients with SIJ abnormalities (%)	Mean age (years)	Male (%)	Type of morphology investigated	Follow-up period	Patient reported outcomes
Krishnamoorthy et al. (2019)	743	187 (25.2%)	33.3 ± 12.2	31.8%	Radiographic SI joint changes	2 years	SIJ group vs. Control: HOS-ADL: 90.6 vs. 95.4, HOS-SS: 77.5 vs. 91.1, mHHS: 84.5 vs. 91.3
Horner et al. (2022)	223	73 (32.7%)	35.6 ± 10.4	18.7%	SI joint pain on history or exam	2 years	SIJ group vs. Control: HOS-ADL: 80.4 vs. 88.0, mHHS: 73.2 vs. 80.0, iHOT-12: 61.7 vs. 73.7
Ferguson et al. (2019)	57	8 (28%) in LO group, 2 (7%) in control group	44.6 (LO group), 44.8 (control)	39.3%	CT grading of SI joint in labral ossification	NR	NR

*HOS-ADL*: hip outcome score-activities of daily living; *HOS-SS*: hip outcome score-sports subscale; *mHHS*: modified harris hip score; *iHOT-12*: international hip outcome tool-12; *LO*: labral ossification *NR*: not reported; *SI*: sacroiliac

### FAI morphology in patients with axial spondyloarthritis

**Fig. 2:**
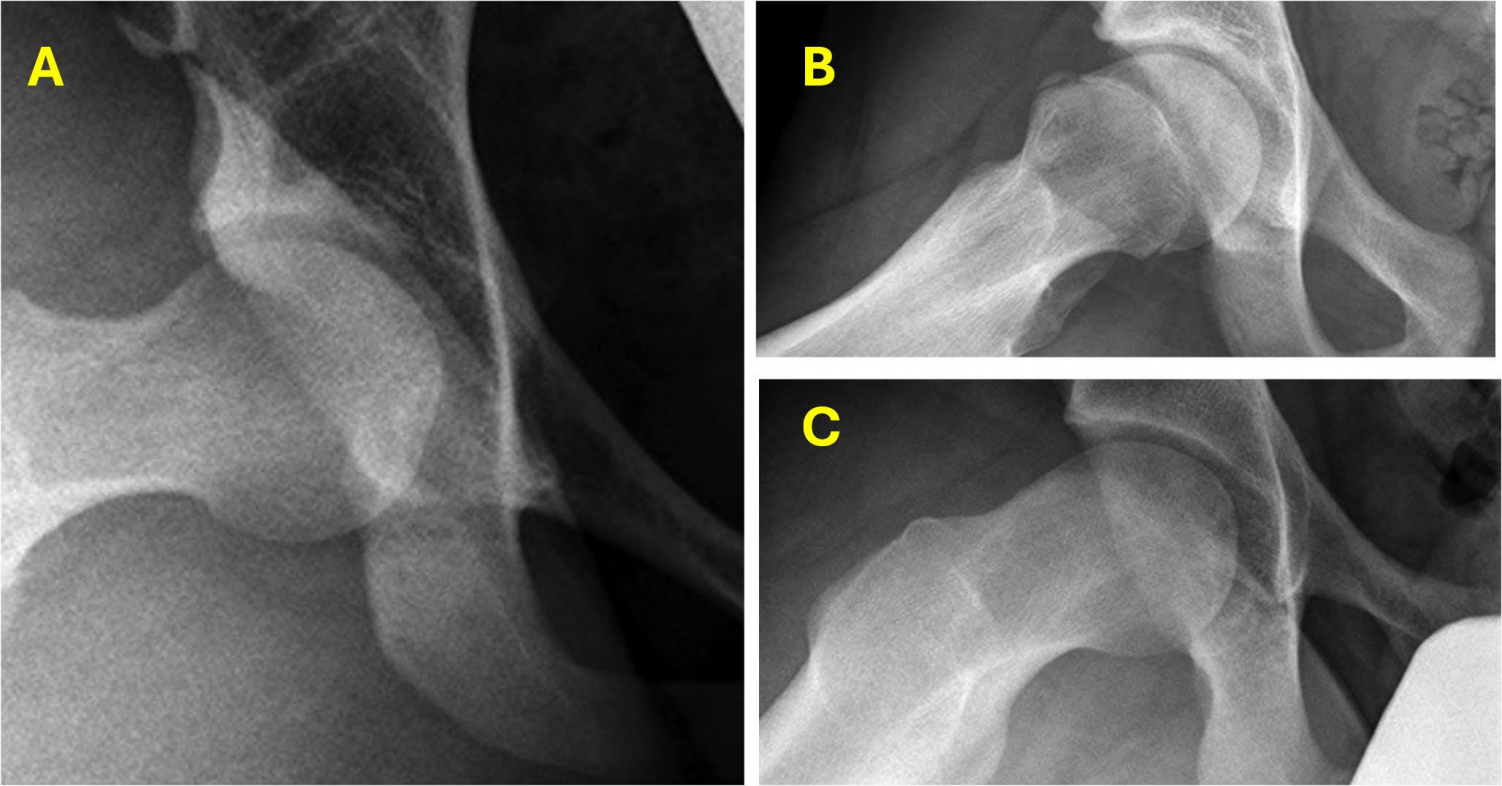
The radiographic images demonstrate three distinct hip presentations. (**A**) normal hip morphology without symptoms, (**B**) a symptomatic hip displaying noninflammatory cam changes, and (**C**) a hip exhibiting the characteristic inflammatory beak formation of the cam deformity, which is frequently observed in patients with underlying inflammatory conditions [12]

Three studies investigated the prevalence and characteristics of FAI morphology in patients with axSpA [11, 13, 27]. Lee et al. observed FAI morphometry in 36.7% of AS patients, with pincer-type being most prevalent (20.6%), followed by combined-type (9.6%) and cam-type (6.5%). The FAI group demonstrated a male predominance (87.2%) and significantly higher sacroiliitis scores compared to non-FAI patients [10]. Combined-type FAI correlated with the highest sacroiliitis scores [11]. Tosun et al. specifically examined cam-type deformity, reporting a higher prevalence of pistol grip deformity in axSpA patients versus controls (20.3% vs. 8.8%, *p* = 0.002) [20]. They identified significant associations between pistol grip deformity and male sex, smokers, and hip arthritis presence. Seth et al., while not focusing exclusively on axSpA, identified a unique “inflammatory beak” cam morphology (Fig. 2) in 8.9% of paediatric FAI patients with various inflammatory conditions, including AS [17]. This morphology was characterised by a negative femoral head-neck offset and increased alpha angles [28].

### Clinical outcomes in patients with FAI and concomitant axial pathologies

Only two studies, totalling 816 patients, reported PROs for FAI patients with concomitant axial pathologies [18, 25]. Both studies utilised the modified Harris Hip Score (mHHS) and the Hip Outcome Score-Activities of Daily Living (HOS-ADL). Krishnamoorthy et al. found significantly lower postoperative scores in patients with SI joint changes compared to those without for both mHHS (84.5 vs. 91.3, *p* < 0.001) and HOS-ADL (90.6 vs. 95.4, *p* = 0.001). Similarly, Horner et al. reported lower postoperative scores in patients with SI joint pain for mHHS (73.2 vs. 80.0, *p* < 0.001) and HOS-ADL (80.4 vs. 88.0, *p* = 0.006). Krishnamoorthy et al. demonstrated that patients without SI joint abnormalities had significantly greater odds of achieving MCID and PASS for multiple outcome measures. Horner et al. reported significantly lower achievement of MCID for HOS-ADL (65.2% vs. 80.5%, *p* = 0.044) and lower achievement of PASS for mHHS (27.5% vs. 45.3%, *p* = 0.030) and iHOT-12 (31.0% vs. 56.0%, *p* = 0.010) in the SI joint pain group.

## Discussion

The main findings of this scoping review reveal that the prevalence of SI joint abnormalities can be as high as 25–28% in patients with FAI. Furthermore, the prevalence of FAI morphology in patients with axial spondyloarthritis can be as high as 20–37%. Finally, patients undergoing hip arthroscopy for FAI with axSpA and/or SI joint abnormalities have lower postoperative outcome scores reported in comparison with those patients who do not have these comorbidities.

A large portion of the literature examined the prevalence of these conditions. Radiological SI joint abnormalities were present in 25.2–28.6% of patients with FAI [18, 24]. Whilst SI joint abnormalities can be found in up to 65% of asymptomatic adults in the general population [29], the patient population in these papers was younger, with mean ages of 32.3 and 44.6 years respectively [18, 24], in contrast to the general population where SI joint abnormalities typically increase with age and are more common in older adults [29]. The prevalence of radiographic FAI morphology in patients with axSpA ranged from 20.3 to 36.7% [11, 13], which needs consideration against the background of axSpA’s male predominance, given that radiographic FAI morphology is found in 20–30% of healthy young adults and up to 60% in male athletic populations [30, 31]. Of note, 77% of patients with SI joint pain exhibited at least one radiological abnormality consistent with FAI [26]. These findings suggest the need for sex and activity-matched controlled studies to determine whether these relationships have clinical significance beyond background prevalence rates. Regarding outcomes, while limited data suggests patients with concomitant FAI and SI joint changes demonstrated lower postoperative clinical scores following hip arthroscopy, specific outcomes for FAI patients with axSpA could not be determined from this review.

Hip arthroscopy has emerged as a less invasive alternative to open hip surgery for treating FAI, offering reduced morbidity and faster recovery [32–34]. However, patient selection remains crucial for optimal outcomes, and identifying reliable predictive factors is essential for setting realistic expectations and guiding rehabilitation. While numerous patient characteristics have been linked to poor outcomes after arthroscopic surgery for FAI, including osteoarthritis, older age, and longer symptom duration [10, 35], and lateral joint space width [36], the influence of underlying inflammatory pathologies like axSpA has been less extensively studied. Exploring the relationship between FAI and inflammatory conditions is a critical step in understanding how these factors interact and affect surgical outcomes. This may necessitate a shift in focus toward new areas of FAI research beyond traditional athletic populations [37] to better serve the broader patient population.

The consistency in reporting elevated prevalence rates across these studies suggests a relationship between FAI and axSpA and/or SI joint involvement. This focus on prevalence in the literature underscores a tangible link between these conditions, establishing a foundation for further investigation. It also highlights the necessity of evaluating SI joint symptoms in patients diagnosed with FAI who are considering arthroscopic treatment.

**Fig. 3:**
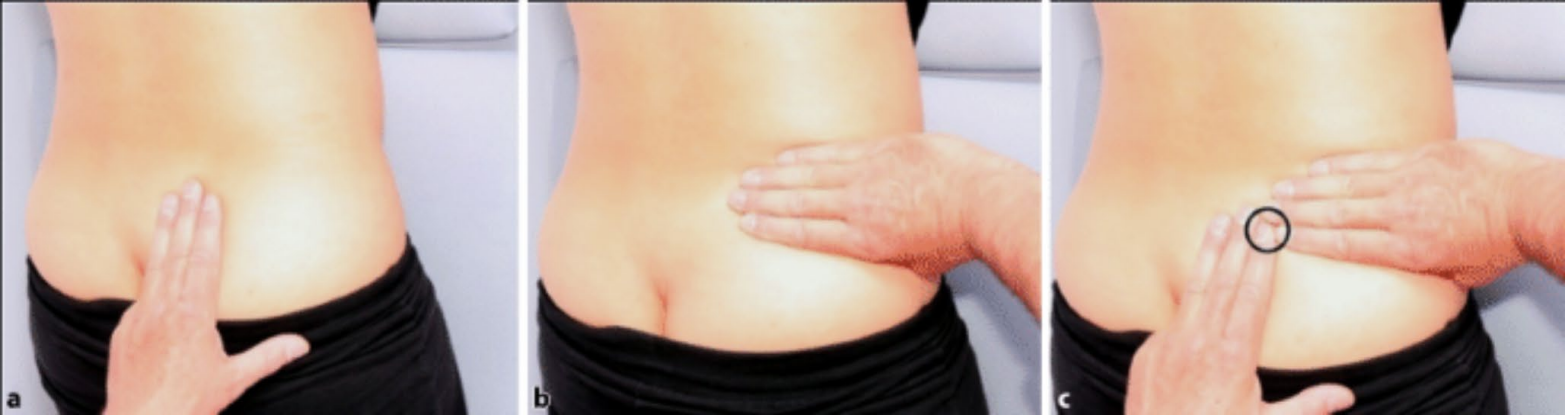
Clinical examination demonstrating the sacroiliac joint pain provocation test (**a**) initial hand placement on ASIS, (**b**) posterior pressure application, and (**c**) target area for pain provocation (black circle) [39]

Assessment of SI joint involvement should include standard history-taking and physical examination, with attention to both subjective indications of SI pain reported by patients and objective tenderness upon SI joint palpation (Fig. 3). SI joint pain can be distinguished from lower back pain if both the thigh thrust and distraction tests are positive, or if one of these tests is positive followed by a positive compression test [26]. While a detailed description of these clinical tests is beyond the scope of this review, their importance in differential diagnosis cannot be overstated. Radiological assessment of SI joint pathology typically follows the New York Criteria [38].

While both FAI and axSpA can affect the hip joint, they do so through distinct mechanisms. FAI represents a mechanical condition characterised by morphological variations in the femoral head-neck junction or acetabulum that lead to abnormal articular contact during hip motion. In contrast, axSpA manifests as a systemic inflammatory disease with the potential to affect large joints through immune-mediated pathways, independent of mechanical factors. This fundamental pathophysiological distinction has significant clinical implications, particularly in diagnostic approaches and therapeutic decision-making. The mechanical impingement in FAI typically produces movement-dependent symptoms and specific patterns of cartilage and labral damage, whilst inflammatory arthritis associated with axSpA presents with characteristic morning stiffness, systemic features, and a different pattern of joint involvement. Understanding these distinct disease patterns becomes crucial in clinical practice, as it influences both diagnostic strategies and treatment algorithms. This understanding underscores the importance of comprehensive evaluation in patients presenting with hip pain, particularly when considering surgical intervention. The presence of inflammatory markers, HLA-B27 status, and specific imaging features can help differentiate between mechanical and inflammatory aetiologies, guiding appropriate specialist referral and treatment selection.

The morphological observations reported by Seth et al. merit particular attention in the context of phenotypic variations across different patient populations. Their identification of a distinct cam morphology in paediatric patients with inflammatory conditions, characterised by a ‘sharp-edged’ head-neck junction, negative femoral offset, and increased alpha angles, represents a potentially significant radiological finding. This unique morphological pattern may serve as an important diagnostic indicator when evaluating young patients with hip pain, particularly in the context of concurrent inflammatory conditions. While the pathophysiological basis for these distinct morphological features remains to be elucidated, their recognition contributes to our understanding of the heterogeneous manifestations of hip pathology and underscores the importance of comprehensive radiological assessment in different patient subgroups.

Perhaps of most relevance to contemporary practice are the clinical outcomes. Our scoping review identified only two studies that specifically explored clinical outcomes in patients with both FAI and SI joint involvement. Horner et al. compared 70 patients with SI joint pain to 150 matched controls undergoing hip arthroscopy for FAI. Although both groups showed significant functional improvement, those with SI joint pain had lower postoperative PRO scores, with HOS-ADL, mHHS, and iHOT-12 scores were reduced compared to controls. In contrast, Krishnamoorthy et al., analysing 743 cases, found that patients with FAI and SI joint abnormalities on imaging had notably poorer outcomes, with a 30% lower success rate at a minimum of 2 years postoperatively compared to those without SI joint changes.

Horner et al. focused on SI joint pain, while Krishnamoorthy et al. examined SI joint changes on imaging, which may not always correlate with symptomatic presentation. This distinction highlights the need for standardised criteria in future studies to define and assess SI joint involvement in patients with FAI. These studies collectively suggest that the presence of SI joint pathology alongside FAI might lead to suboptimal postoperative results following hip arthroscopy, even in the absence of apparent SI joint related symptoms. These findings are further supported by a recent systematic review by Lee et al. [20], which examined the impact of low back pain on hip arthroscopy outcomes. Their review, encompassing 14 studies, found that over 50% of the studies reported worse outcomes after hip arthroscopy in patients with low back pain compared to those without. These findings have important clinical implications, emphasising the need for comprehensive evaluation and careful patient selection for hip arthroscopy in individuals with FAI and potential SI joint involvement.

Our review identified methodological variations in how these conditions were studied. FAI-focused studies primarily examined SI joint abnormalities or sacroiliitis as isolated findings, as demonstrated by Krishnamoorthy et al. and Horner et al. [12, 15]. Studies of axSpA cohorts included assessments of FAI morphology [16, 36], though without adjusting for demographic factors such as age and sex distribution. This heterogeneity in study approaches limits our ability to draw robust conclusions about relationships between these conditions. Future research would benefit from prospective cohort studies employing standardised diagnostic criteria, appropriate demographic matching, and comprehensive outcome measures to better understand the clinical implications of concurrent FAI and SI joint pathology.

### Limitations

This scoping review has several limitations that must be considered. First, the paucity of studies directly investigating the pathophysiological link between FAI and axSpA limits our ability to draw definitive conclusions about the relationship between FAI and axSpA. Second, the variability in defining and assessing SI joint involvement across studies makes it challenging to synthesise findings consistently. Third, the lack of longitudinal studies restricts our understanding of the temporal relationship between these conditions. Fourth, most studies lacked appropriate control groups, making it difficult to determine if the observed relationships exceed background prevalence rates in matched populations. Despite these limitations, a key strength of this review is its comprehensive mapping of the current literature landscape across prevalence studies, clinical outcome assessments, and proposed pathophysiological mechanisms. This broad perspective effectively highlights the gaps in our understanding and provides a clear direction for future research.

## Conclusion

Over a quarter of patients with FAI may have concurrent SI joint abnormalities. This underscores the importance of assessing the spine and excluding SI joint pathology, axSpA, and other sources of pain in patients presenting with FAI. The potential for poorer post-operative outcomes in patients with concomitant SI joint abnormalities emphasises the need for refined surgical selection criteria and careful management of patient expectations.

## Data Availability

All data supporting the findings of this study are available within the paper and its Supplementary Information.

## Abbreviations

AS: Ankylosing Spondylitis
axspa: Axial Spondyloarthritis
CT: Computed Tomography
FAI: Femoroacetabular Impingement
HOS-ADL: Hip Outcome Score-Activities of Daily Living
iHOT-12: International Hip Outcome Tool-12
MCID: Minimal Clinically Important Difference
mHHS: Modified Harris Hip Score
MRI: Magnetic Resonance Imaging
OA: Osteoarthritis
PASS: Patient Acceptable Symptomatic State
PROs: Patient-Reported Outcomes
PRISMA: Preferred Reporting Items for Systematic Reviews and Meta-Analyses
SI: Sacroiliac
